# Diabetes consequences in the fetus liver of the non-obese diabetic mice

**DOI:** 10.1038/nutd.2017.7

**Published:** 2017-03-20

**Authors:** M B Aires, A C V dos Santos, M S Kubrusly, A C de Lima Luna, L A C D'Albuquerque, D A Maria

**Affiliations:** 1 Department of Morphology, Federal University of Sergipe, Sao Cristovao, Sergipe, Brazil; 2 Discipline of Liver Transplant and Digestive Organs (LIM-37), Faculty of Medicine, University of Sao Paulo, Sao Paulo, Brazil; 3 Laboratory of Biochemistry and Biophysics, Butantan Institute, Sao Paulo, Brazil

## Abstract

DM type 1 (T1D) incidence is increasing around 3% every year and represents risks for maternal and fetal health. The objective of this study was to explore the effects of diabetes on fetus liver cells in non-obese diabetic (NOD) mice. Hyperglycemic NOD (HNOD), normoglycemic NOD (NNOD) and BALB/c females were used for mating, and the fetus livers were collected at 19.5 gestation day (gd). HNOD group had reduced fetal weight (989.5±68.32 vs 1290±57.39 mg BALB/c, *P*<0.05) at 19.5 gd and higher glycemia (516.66±28.86 mg dl^−1^, *P*<0.001) at both 0.5 gd and 19.5 gd compared to other groups. The protein expression of albumin (ALB) was significantly reduced in HNOD group (0.9±0.2 vs 3.36±0.36 NNOD *P*<0.01, vs 14.1±0.49 BALB/c *P*<0.001). Reduced gene expression of ALB (1.34±0.12 vs 5.53±0.89 NNOD and 5.23±0.71 BALB/c, *P*<0.05), Hepatic Nuclear Factor-4 alpha (HNF-4α) (0.69±0.1 vs 3.66±0.36 NNOD, *P*<0.05) and miR-122 (0.27±0,10 vs 0.88±0.15 NNOD, *P*<0.05) was present in HNOD group. No difference for alpha-Fetoprotein (AFP) and gene expression was observed. In conclusion, our findings show the impacts of T1D on the expression of ALB, AFP, HNF-4α and miR-122 in fetus liver cells by using NNOD and HNOD mice.

## Introduction

Diabetes mellitus (DM) is considered a worldwide public health problem and considering all types of diabetes; there was an estimated 184 million diabetic women in 2013, a number expected to rise to 288 million by 2030.^1^ Although DM type 1 (T1D) is less frequent, the data from International Diabetes Federation pointed an increase around 3% every year. In 2015, Brazil was found to have the third highest number of children with this form of diabetes in the world.^1^ Consequently, there will be an increase in T1D women at reproductive age who will be subject to diabetic complications during pregnancy if they are left poorly controlled.

Rodent diabetic experimental models have been used to study the impacts of maternal diabetes in fetal development; however, few studies focused on fetal liver gene and protein expression. The non-obese diabetic (NOD) mouse is a well-characterized model of autoimmune T1D that develops spontaneous pre-gestational diabetes and has advantages as stable hyperglycemia throughout gestation and lack of chemical destruction of β-cells.^2, 3^

Although the liver has a high regenerative capacity, failure can occur due to massive death of hepatocytes as a result of acute, chronic and hereditary diseases. MicroRNA-122 (miR-122) is a highly abundant and liver-specific miRNA that accounts for 70% of the total liver miRNA population.^4^ As a result, miR-122 is crucial for liver development, differentiation, homeostasis and functions.^5^ Evidences indicate that the activation of miR-122 plays an important role in guidance hepatocyte differentiation and maturation *in vitro* and *in vivo*.^6, 7^

The miR-122 expression is driven by liver-enriched transcription factors, including hepatocyte nuclear factor (HNF) 6 and 4α that also fine-tune miR-122 dosage during liver development *in vivo*.^6, 7, 8^ Particularly, the expression of HNF-4α is upregulated during overexpression of miR-122^7^ and zebrafish^6^ hepatocyte, and not cholangiocyte differentiation.

In view of the importance of diabetes for maternal and neonatal health, the objective of the present study was to assess the influence of diabetes on the expression of albumin (ALB), alpha-Fetoprotein (AFP), Hepatic Nuclear Factor-4 alpha (HNF-4α) and miR-122 by liver fetal cells from hyperglycemic and normoglycemic NOD mice at 19, 5 day of gestation.

## Methods

### Animals

All procedures were performed under the guidance of the Committee for Animal Experimentation of Butantan Institute - CEUAIB (Protocol. 1239/14). Female NOD/Unib were used to form the experimental groups: hyperglycemic (HNOD group, non-fasting glucose⩾270.0 mg dl^−1^, *n*=6, 22±2 g) and normoglycemic (NNOD group, non-fasting glucose <180.0 mg dl^−1^, *n*=6, 25±2 g) females. Male normoglycemic NOD (non-fasting glucose <180.0 mg dl^−1^, *n*=8, 30±2 g) were used for mating. Pregnant BALB/c normoglycemic females (*n*=6, 25±2 g) were used as an additional group. The onset of pregnancy was determined by detection of the vaginal copulation plug and was designated 0.5 gestation day (gd). On 19.5 gd, the animals were anaesthetized with xylazine (20 mg kg^−1^) and ketamine (80 mg kg^−1^), exsanguinated and laparotomized to remove the uterine horns for collection of fetuses. The sample size was estimated in the pilot study by the Select Statistical website (https://select-statistics.co.uk/calculators/sample-size-calculator-population-mean/). Randomization was used only to allocate females in the BALB/c group because the selection of females for NOD groups was based on glycemia.

Liver samples were dissected and frozen for protein and gene expression analysis. All experiments were replicated at least twice, and blinded analysis was performed when assessing the outcome.

### Flow cytometry

Liver samples were dissected and mechanically dissociated, filtered through 25 mm sterile filters, added freezing solution and kept in a −70 °C freezer. Fluorescence-activated cell sorting analysis was performed as reported previously^9^ with primary antibodies: goat polyclonal anti-ALB (sc-8108, Santa Cruz, CA, USA), goat polyclonal anti-AFP (sc-46293, Santa Cruz, CA, USA) at 4 °C overnight. The corresponding isotype antibody was used as negative control and as a secondary antibody was used rabbit anti-goat IgG: Alexa 594 (Immuny, Campinas, Brazil).

### RNA isolation

Total RNA was isolated using Direct-zol RNA MiniPrep kit (Zymo Research, Irvine, CA, USA) according to manufacturer's protocol instructions from the frozen liver tissues from all groups. The RNA quality and total RNA concentration were performed as reported previously.^10^ Samples were kept at –80 °C until processing by quantitative RT-PCR (qRT-PCR).

### miRNA isolation and amplification

To obtain reliable levels of miRNA, total RNA was isolated from frozen liver tissues using a miRNA Isolation Kit (Ambion, Austin, TX, USA). cDNA were generated using the following kits: TaqMan MicroRNA Reverse Transcription (RT) and TaqMan Small RNA Assays (Applied Biosystems, Foster City, CA, USA) in a StepOne Plus Thermocycler (Applied Biosystems, CA, USA) for both RT and qRT-PCR reactions. For qPCR amplification reaction of miR-122, the product of RT reaction was mixed with the respective assays (miR-122 and U6) and 2 × TaqMan Universal PCR Master Mix II in a 96-well plate according to manufacturer's recommendations. The reactions were conducted in duplicate, and U6 was used as an endogenous control. The miR-122 fold expression was calculated by application of 2^−ΔΔ^Ct method.^11^

### Quantitative RT-PCR analysis

qRT-PCR analysis to assess gene expression of Albumin (ALB), Alpha-Fetoprotein (AFP) and Hepatic Nuclear Factor-4 (HNF-4α) was performed in the Rotor-Gene RG-3000 Thermocycler (Corbett Research, Sidney, Australia) using SuperScript III Platinum^®^ SYBR Green One-Step qRT-PCR kit (Applied Biosystems Carlsbad, CA, USA), according to manufacturer's recommendations. GAPDH mRNA was used as an endogenous control. The following primer sets were used: ALB 5′-CCCACTAGCCTCTGGCAAAA-3′ 5′-ACACACCCCTGGAAAAAGCA-3′ AFP 5′-AGGAGGAGTGCTTCCAGACA-3′ 5′-TGGTTGTTGCCTGGAGGTTT-3′ HNF-4α 5′-TACCTTCCTCCGCCATCTGA-3′ 5′-TCCTACCCTCTGCCTTACCC-3′ GAPDH 5′-ACTGAGCAAGAGAGGCCCTA-3′ 5′-TATGGGGGTCTGGGATGGAA-3′. The relative expression level of each gene was determined relative to the GAPDH transcript by the comparative ΔΔCt method.^11^

### Statistical analysis

The data analysis was performed by comparison with parametric distribution of the groups using Student *t*-test or analysis of variance (ANOVA) followed by Tukey–Kramer multiple comparison test. The data was analyzed using the Prism 5.0 Statistical Software package (GraphPad, San Diego, CA, USA) and are presented as the mean±s.e.m, a value of *P*<0.05 was considered significant.

## Results

### Maternal glycemia and fetal weight

In NNOD and BALB/c groups the female blood glucose levels were similar at 0.5 gd (134.08±14.67; 93.50±10.81 mg dl^−1^) and 19.5 gd (118.08±6.84; 102.5±9.67 mg dl^−1^). In the HNOD group, a significantly higher glycemia (516.66±28.86 mg dl^−1^, *P*<0.001) was found at both 0.5 gd and 19.5 gd compared to other groups. The fetal weight was reduced in the HNOD group (989.5±68.32 mg, *P*<0.05) compared to the BALB/c group (1290±57.39 mg, *P*<0.05).

### Expression of ALB and AFP by fetal liver cells

**Figure 1:**
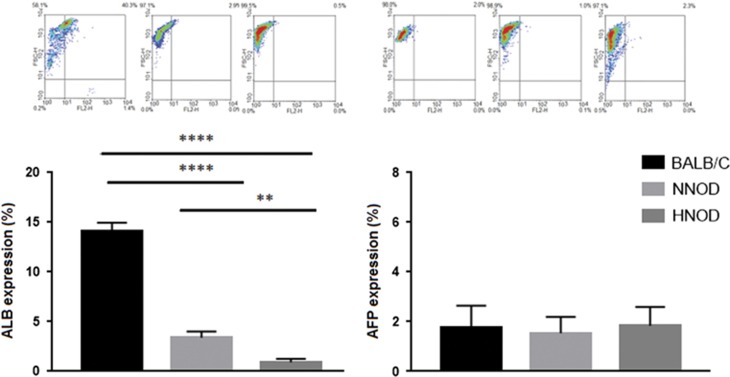
Representative dot plot of fetus liver cells from BALB/c, NNOD and HNOD groups at 19.5 dg stained with Albumin (ALB) and Alpha-Fetoprotein (AFP) markers. The histogram show increased expression of ALB in HNOD group compared to NNOD and BALB/c groups. The expression of AFP was not different between groups. Results represent SEM of liver cells collected from fetus livers per animal of control (*n*=6), NNOD (*n*=6) and HNOD groups (*n*=6) analyzed in duplicate, **P<0.01, ****P<0.0001.

The expressions of ALB and AFP were detected on fetal liver cells in all groups (Figure 1). The expression of ALB was significantly reduced in HNOD group (0.9±0.2) compared to NNOD (3.36±0.36, *P*<0.01) and BALB/c (14.1±0.49, *P*<0.0001). The expression of AFP was not different between groups (1.75±0.44 BALB/c, 1.53±0.38 NNOD, 1.83±0.44 HNOD).

### Gene expression of ALB, AFP, HNF-4α and miR-122

**Figure 2:**
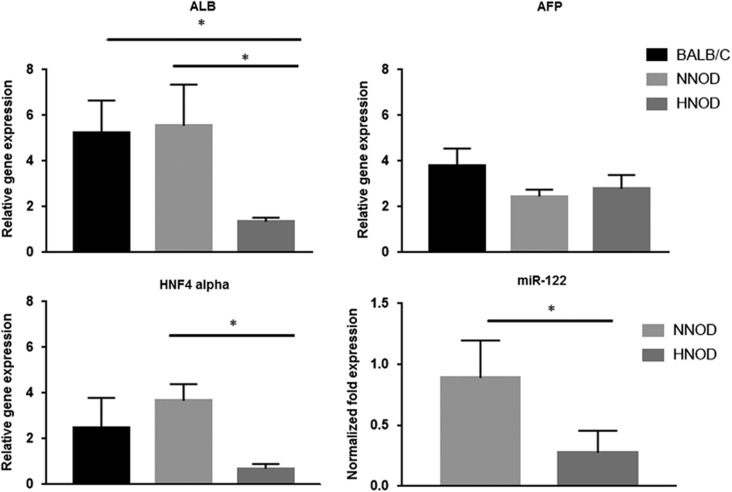
Albumin (ALB), alpha-fetoprotein (AFP), Hepatic nuclear factor-4 (HNF-4α) mRNAs and miR-122 expression levels in fetus liver cells of BALB/c, NNOD and HNOD groups at 19.5 dg. Each NNOD and HNOD sample was normalized to a BALB/c sample and, the expression level of ALB, AFP, HNF-4α was calculated by application of ΔΔCt method and the miR-122 fold expression by 2^−ΔΔ^Ct method. The data represent SEM of liver cells collected from fetus livers per animal of control (*n*=6), NNOD (*n*=6) and HNOD groups (*n*=6) analyzed in duplicate, *P<0.05.

Reduced gene expression of ALB was present in HNOD group (1.34±0.12, *P*<0.05) compared to NNOD (5.53±0.89) and BALB/c (5.23±0.71) groups (Figure 2). Low expression of HNF-4α was observed in HNOD group (0.69±0.1 vs 3.66±0.36 NNOD, *P*<0.05). The gene expression of AFP was not different between groups (3.80±0.43 BALB/c, 2.46±0.17 NNOD, 2.79±0.34 HNOD) (Figure 2). The miR-122 expression was significantly reduced in HNOD (0.27±0.10, *P*<0.05) compared to NNOD (0.88±0.15) (Figure 2).

## Discussion

Maternal diabetes is still a very important cause of pregnancy complications including fetal defects. The NOD mouse is one of the most commonly used animals that spontaneously develop T1D^12^ and therefore, an important model for this condition in human pregnancy. Moreover, this study is the first to describe the influence of diabetes on the expression of hepatic markers (ALB, AFP), HNF-4α and miR-122 in NOD fetus liver.

In our study, the hyperglycemia in HNOD animals at 0.5 gd and 19.5 gd was characteristic of severe diabetes and together with small litter size, the results agree with the studies of Burke and co-workers.^13^ The reduction of fetal weight observed in HNOD mice was similar to that found in intrauterine growth restriction rodent models for severe diabetes.^14^

The decrease of miR-122 in HNOD animals could be related to the reduction of HNF-4α by hepatic liver cells. Some works have evidenced that miR-122 inhibition repressed the expression of many genes and that the expression of HNF-4α could be directly or indirectly (via HNF-6) affected by miR-122.^6^ One interesting point to be considered is that the miR-122 expression is regulated in part by HNF-1α and HNF-4α in cultured hepatocellular cancer-derived cells^7^ and adult liver^15^ raising the possibility that many liver-enriched transcription factors (LETFs) positively feedback on miR-122 expression to control hepatocyte differentiation.^6^ Whether this happens in NOD fetal liver under hyperglycemia should be investigated.

The miR-122 plays a central role in liver development, differentiation, homeostasis and functions. It seems that it controls the proper balance between cell proliferation and differentiation in both hepatocyte and cholangiocyte lineages influencing terminal liver differentiation.^5^ Indeed, the reduction of ALB gene and protein expression in fetal liver HNOD cells could indicate a reduction of hepatoblast maturation or terminal differentiation, besides the AFP normal expression. The reduction of HNF-4α gene expression in HNOD group could also be correlated with the reduction of ALB expression as blockage of HNF-4α decrease the expression hepatocyte-specific proteins such as apolipoproteins and albumin, suggesting its critical role for hepatocyte fate determination in mice liver.^16^

Other possible miR-122 functions in the adult liver as regulation of cholesterol and fatty acid metabolism should be investigated in fetal liver cells under diabetes condition. At the same manner, the role of HNF-4α in the determination of cell morphology^17^ is unknown in NOD fetal liver cells. In diabetes experimental condition, only one work described increased HNF-4α gene expression in adult liver cells of alloxan-induced diabetic rats.^18^ However, experimental model and life stage differences could be determinant in gene expression responses under hyperglycemia, which makes comparisons among studies difficult.

Epigenetics effects of maternal diabetes on the offspring have been identified in animal streptozotocin-induced maternal diabetes, in which histone H3 and H4 acetylation of embryonal genes was implicated in neural tube defects.^19^ Therefore, the liver could be a target for epigenetic modifications under the intrauterine hyperglycemic environment, which might contribute to liver metabolic programming of insulin resistance and diabetes during postnatal life, as also observed in overnutrition fetal environment.^20^

In conclusion, results of this study showed altered expression of ALB, HNF-4α and miR-122 in HNOD fetus liver. Therefore, many cellular mechanisms during the differentiation and maturation of liver cells may be affected by the diabetes condition leading to possible postnatal and adult liver diseases.
